# A large iliopsoas abscess due to migration of ureteral stent: a case report

**DOI:** 10.3389/fsurg.2026.1975183

**Published:** 2026-09-29

**Authors:** Haipeng Sun, Xiande Cao, Yongtao Su, Siyuan Liu

**Affiliations:** 1 The First Clinical Medical College, Shandong University of Traditional Chinese Medicine, Jinan, Shandong, China; 2 Affiliated Hospital of Shandong University of Traditional Chinese Medicine, Jinan, Shandong, China; 3 Shandong University of Traditional Chinese Medicine, Jinan, Shandong, China

**Keywords:** *Candida tropicalis*, case report, iliopsoas abscess, percutaneous drainage, ureteral stent migration

## Abstract

**Background:**

Iliopsoas abscess (IPA) is a rare but potentially life-threatening condition. Due to its nonspecific clinical presentation and low incidence, IPA often leads to diagnostic delays and suboptimal initial management. Secondary IPA originating from urinary tract complications is extremely rare, and IPA induced by ureteral stent migration appears to be exceptionally uncommon. Fungal IPA caused by *Candida tropicalis* is also a scarcely reported clinical entity, especially in immunocompromised patients with long-term rheumatoid arthritis (RA).

**Case presentation:**

We report a 63-year-old woman with a history of rheumatoid arthritis (RA) and prior colon cancer surgery that involved partial ureteral resection and ureteral stent implantation 6 months ago. The patient later developed IPA, which was successfully managed with dual ultrasound-guided percutaneous drainage, ureteral stent exchange, and systemic antimicrobial therapy. Cultures of the drained abscess and urine both yielded *C. tropicalis*; the drainage-fluid creatinine (5,334 μmol/L) was consistent with urine, suggesting that the IPA likely originated from infected urine leaking from the ureteral anastomosis due to migration of the ureteral stent. The patient was eventually discharged without further complications.

**Conclusion:**

Ultrasound-guided percutaneous drainage is a safe and effective treatment modality for IPA. This case highlights that ureteral stent migration, though typically managed uneventfully, may precipitate severe septic sequelae including IPA, particularly in immunocompromised patients.

## Introduction

IPA is an uncommon suppurative infection of the iliopsoas muscle compartment, classified etiologically into primary forms (arising from hematogenous or lymphatic dissemination of pathogens) and secondary forms (resulting from contiguous spread or direct extension from an adjacent infectious focus) (1). Several host factors predispose to its development, including diabetes mellitus, immunosuppression, intravenous drug use (2). Adverse outcomes, by contrast, are associated with delayed diagnosis, sepsis at presentation, and incomplete source control (2, 3).

The microbiological profile of IPA mirrors its primary source: genitourinary-origin IPA is most often driven by Enterobacteriaceae, especially *Escherichia coli*, with *Klebsiella* spp., enterococci and anaerobes also described; gastrointestinal-origin IPA is typically driven by enteric organisms such as *E. coli* and *Bacteroides fragilis*; and hematogenous IPA is commonly due to *Staphylococcus aureus* (4). Fungal etiologies such as *Candida* are distinctly uncommon in IPA and have been reported mainly as isolated case reports in immunocompromised hosts (5).

Non-genitourinary IPA is principally treated with prompt source control via image-guided percutaneous drainage plus pathogen-directed antimicrobial therapy, open surgery being reserved for gas-forming or multilocular collections in which percutaneous drainage is inadequate (6–8); in contrast, genitourinary-origin IPA is uncommon and lacks standardized therapeutic protocols (1, 3), and its management—whether by image-guided percutaneous drainage, retroperitoneal laparoscopic drainage, or open surgical drainage—is dictated by abscess characteristics and the patient's general condition (7–9). Since accurate identification of the primary infectious source is central to both diagnosis and therapy, the iatrogenic urological precipitants of IPA merit emphasis.

Ureteral stent migration is a recognized complication of indwelling double-J stents, reported in approximately 1%–2% of cases overall (10, 11). Reported predisposing factors include prolonged dwell time and inappropriate stent length (10). Migration can precipitate serious sequelae, including ureteral perforation, luminal obstruction with upstream hydronephrosis, urinary leakage and fistula formation, and peri-stent infection progressing to pyelonephritis or urosepsis (10, 11). This report describes a case of large IPA secondary to ureteral stent migration, which was successfully treated with percutaneous drainage combined with stent exchange.

## Case presentation

A 63-year-old female with a medical history of RA and colon cancer surgery—complicated by partial ureteral resection and ureteral stent placement 6 months prior—presented to the emergency department. She reported a 7-day history of intermittent right lumbar pain and fever. Her regular medications included oral ibuprofen 400 mg three times daily, methotrexate 15 mg once weekly, and prednisone 7.5 mg once daily. During the acute infection, ibuprofen and methotrexate were discontinued, whereas prednisone (7.5 mg/day) was continued unchanged to avoid adrenal insufficiency. Upon admission, vital signs revealed a temperature of 39.3 °C, blood pressure of 94/71 mmHg, and a pulse of 100 beats per minute. Physical examination revealed right costovertebral angle tenderness and tenderness in the right lower quadrant, without rebound tenderness or guarding. Notably, the patient exhibited an inability to extend the right hip while supine, accompanied by tenderness in the right iliac region. Laboratory investigations yielded the following results: leukocyte count, 13.71 × 10⁹/L [normal range (NR): 3.5–9.5 × 10⁹/L]; hemoglobin, 75 g/L (NR: 115–150 g/L); platelet count, 105 × 10⁹/L (NR: 125–350 × 10⁹/L); and C-reactive protein, >200 mg/L (NR: 0–10 mg/L). Urinalysis demonstrated 567.3 p/μL (NR: 0–9 p/μL) white blood cells and 38.3 p/μL (NR: 0–5 p/μL) red blood cells, with no casts and negative nitrites. A urine specimen was sent for culture on admission, which subsequently grew *C. tropicalis* (result available on hospital day 3). Blood cultures were sterile, and no intravascular catheter (e.g., central venous or peripherally inserted central catheter) was present; HIV serology, CA19-9 and CEA levels were all within normal limits, and stool routine examination was unremarkable. An abdominal contrast-enhanced CT scan performed post-admission depicted right iliopsoas muscle swelling, characterized by a long, spindle-shaped mass extending from the psoas major muscle into the retroperitoneal space superior and lateral to the right ilium. Furthermore, right ureteral and renal pelvic dilation accompanied by hydronephrosis was observed (Figure 1A), with the distal tip of the ureteral stent having migrated proximally within the ureter (Figure 1B). The diagnosis of iliopsoas abscess was suspected, attributed to the proximal migration of the ureteral stent.

**Figure 1:**
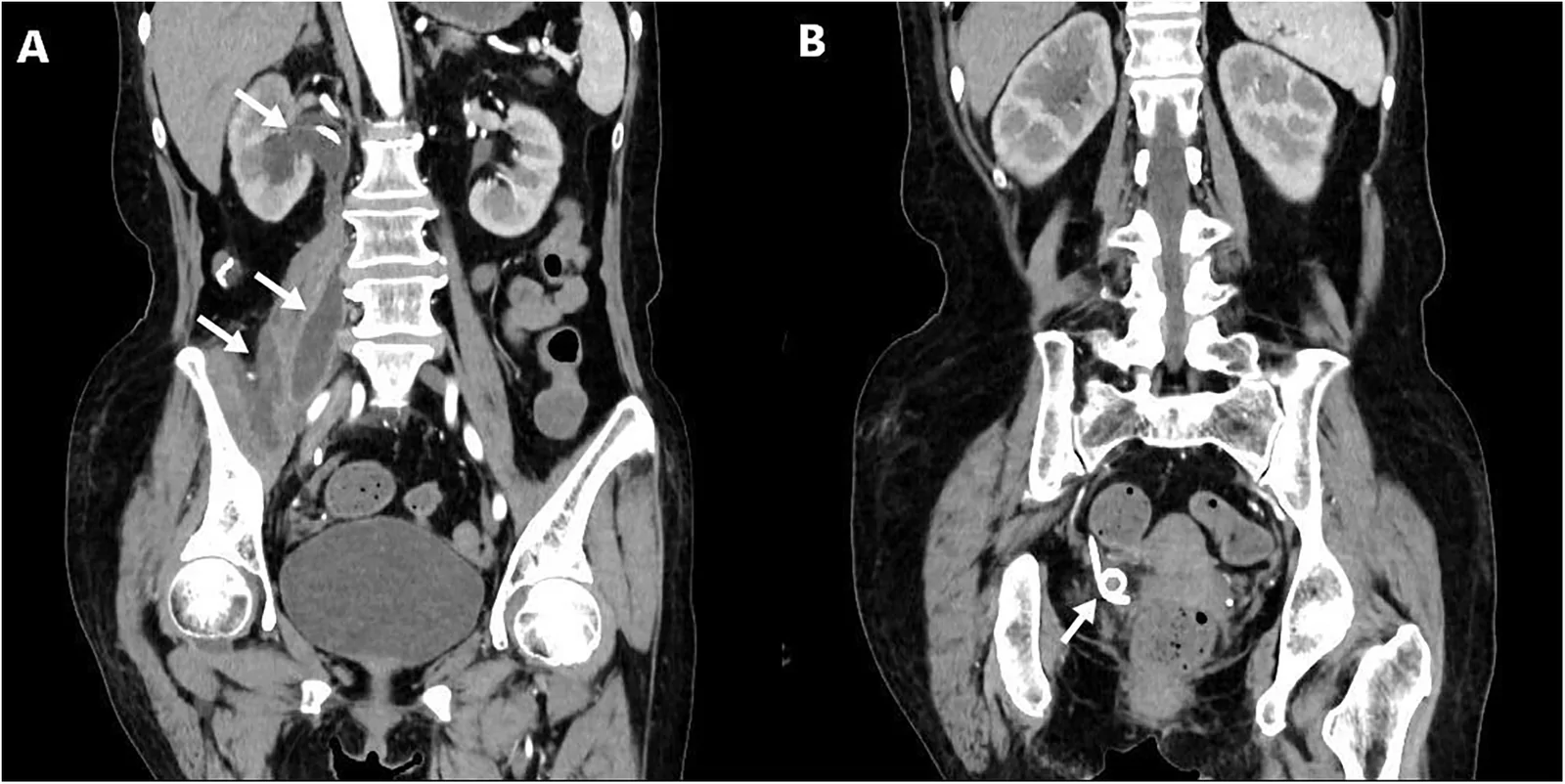
Abdominal contrast-enhanced CT demonstrating the iliopsoas abscess and ureteral stent migration. **(A)** Coronal CT image showing right iliopsoas muscle swelling, characterized by a long, spindle-shaped mass extending from the psoas major muscle into the retroperitoneal space superior and lateral to the right ilium, with right ureteral and renal pelvic dilation and hydronephrosis (arrow). **(B)** The distal tip of the ureteral stent has migrated proximally within the ureter (arrow).

This migration may have resulted in ureteral dilation and hydronephrosis, leading to urine leakage at the previous ureteral anastomosis site and subsequent infection of the retroperitoneal space.

Initial treatment strategies for the IPA were formulated. First, ultrasound-guided percutaneous drainage was performed via a stab incision at the anterior axillary line superior to the ilium. After abscess evacuation, an 8-French pigtail catheter was placed within the abscess cavity of the psoas major muscle (Figure 2A). Microbial culture (direct plating under 5% CO_2_) of the drained fluid identified *C. tropicalis* (result reported 2 days after drainage), concordant with the urine isolate. Pending culture results, empirical antibacterial coverage with cefoperazone-sulbactam 1.5 g every 12 h was administered; once cultures yielded only *C. tropicalis* without bacterial growth, this empirical agent was discontinued and, according to the antibiogram (summarized in Supplementary Table 2), targeted antifungal therapy with intravenous fluconazole 400 mg once daily was initiated. Notably, the catheter yielded continuous large-volume pale-yellow drainage of approximately 400–600 mL daily, with a measured drainage-fluid creatinine level of 5,334 μmol/L—far exceeding the normal serum range (44–106 μmol/L) and consistent with urine, indicating that the abscess cavity contained extravasated urine. Right ureteral stent exchange was performed on day four after initial abscess drainage, after which the pale-yellow drainage completely ceased, indicating that the leak arose from the urinary tract rather than the bowel. This finding suggested that the fluid represented urine leakage from the ureteral anastomosis secondary to stent migration. Despite adequate IPA drainage and stent exchange, right iliac tenderness and restricted right hip motion persisted for 6 days. Follow-up abdominal CT demonstrated interval reduction in the size of the psoas major abscess and decreased surrounding swelling, whereas persistent swelling and new intermuscular abscess formation were observed in the right iliacus muscle (Figure 2B). Accordingly, a second 8-French pigtail catheter was inserted into the iliacus abscess cavity via a lateral iliac approach under ultrasound guidance (Figure 3). Fistulography performed during the procedure confirmed communication between the psoas major and iliacus abscess cavities (Figure 4A). The dual cavities were irrigated with dilute iodine solution through the two indwelling catheters. The patient's right iliac symptoms resolved within 3 days. The drainage tubes were removed after purulent discharge resolved. Intravenous fluconazole had by then been administered for a total of 2 weeks and was discontinued 2 days before discharge, with no oral antifungal step-down prescribed. The patient was discharged without further complications. A 1-month follow-up CT scan revealed near-normal morphology of the iliopsoas muscle and right kidney, with no residual abscess collection (Figure 4B). The ureteral stent was removed 2 months after discharge, and the patient remained recurrence-free at the 6-month follow-up.

**Figure 2:**
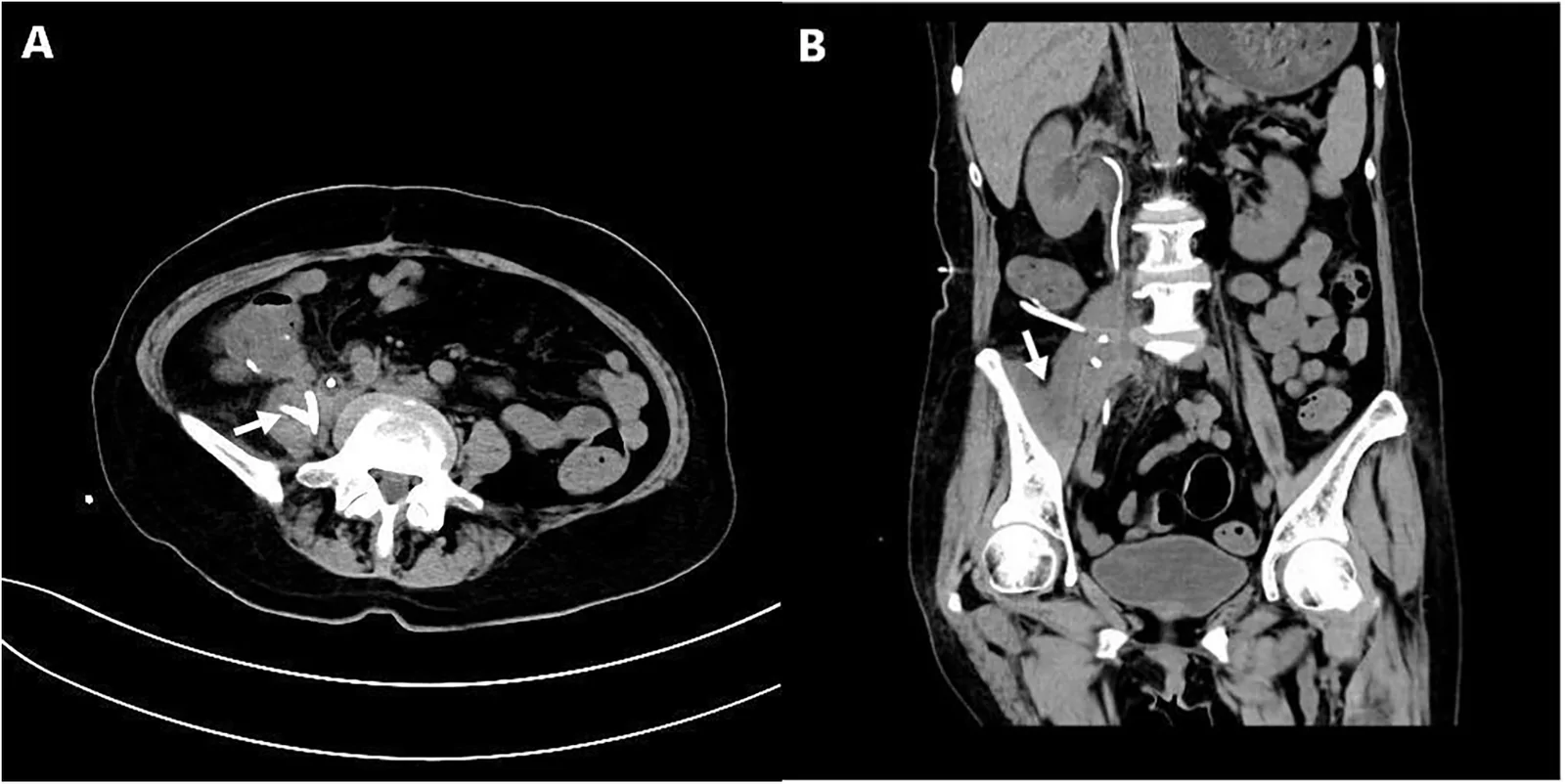
Percutaneous drainage and follow-up CT of the iliopsoas abscess. **(A)** Ultrasound-guided placement of an 8-French pigtail catheter within the abscess cavity of the psoas major muscle (arrow). **(B)** Follow-up abdominal CT demonstrating interval reduction in the size of the psoas major abscess and decreased surrounding swelling, whereas persistent swelling and new intermuscular abscess formation were observed in the right iliacus muscle (arrow).

**Figure 3:**
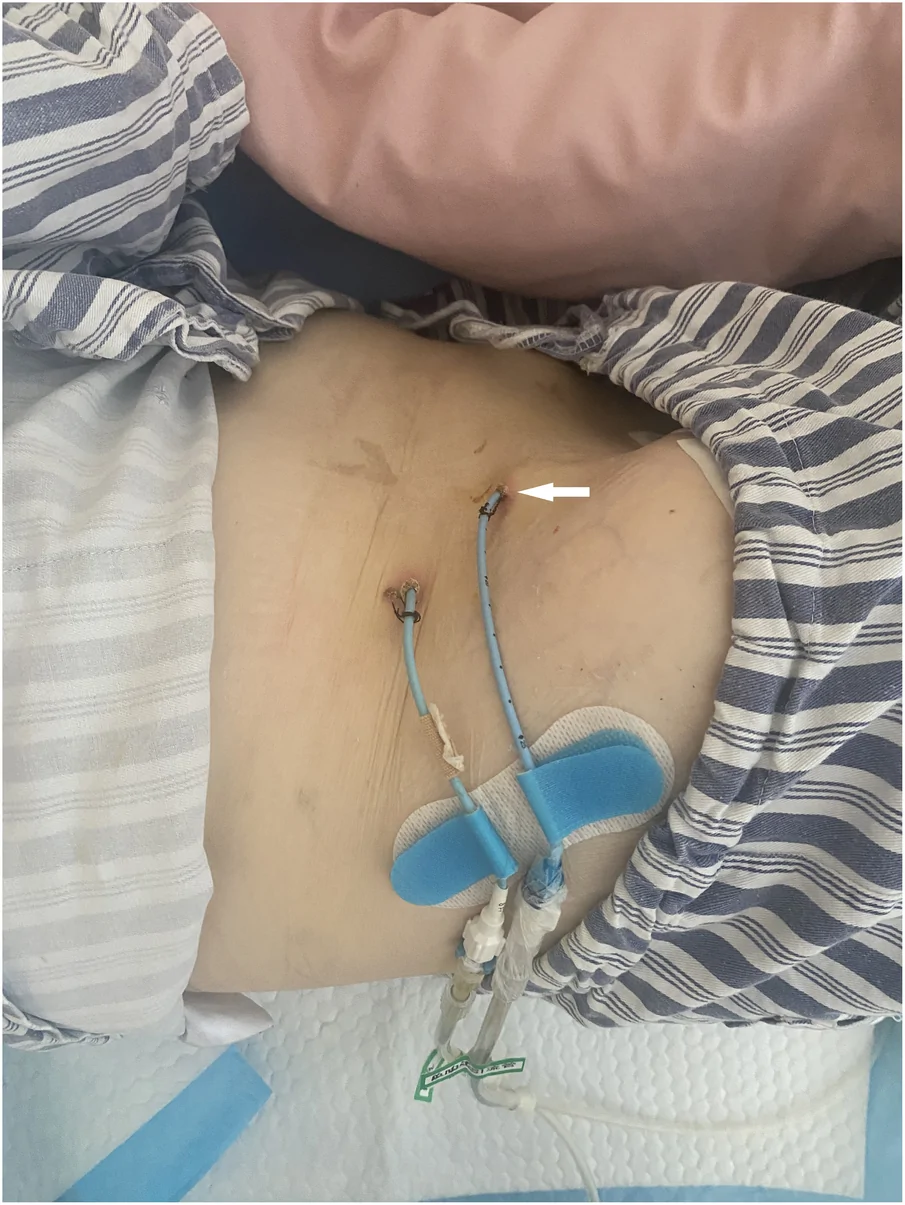
Ultrasound-guided placement of a second 8-French pigtail catheter into the iliacus abscess cavity via a lateral iliac approach (arrow).

**Figure 4:**
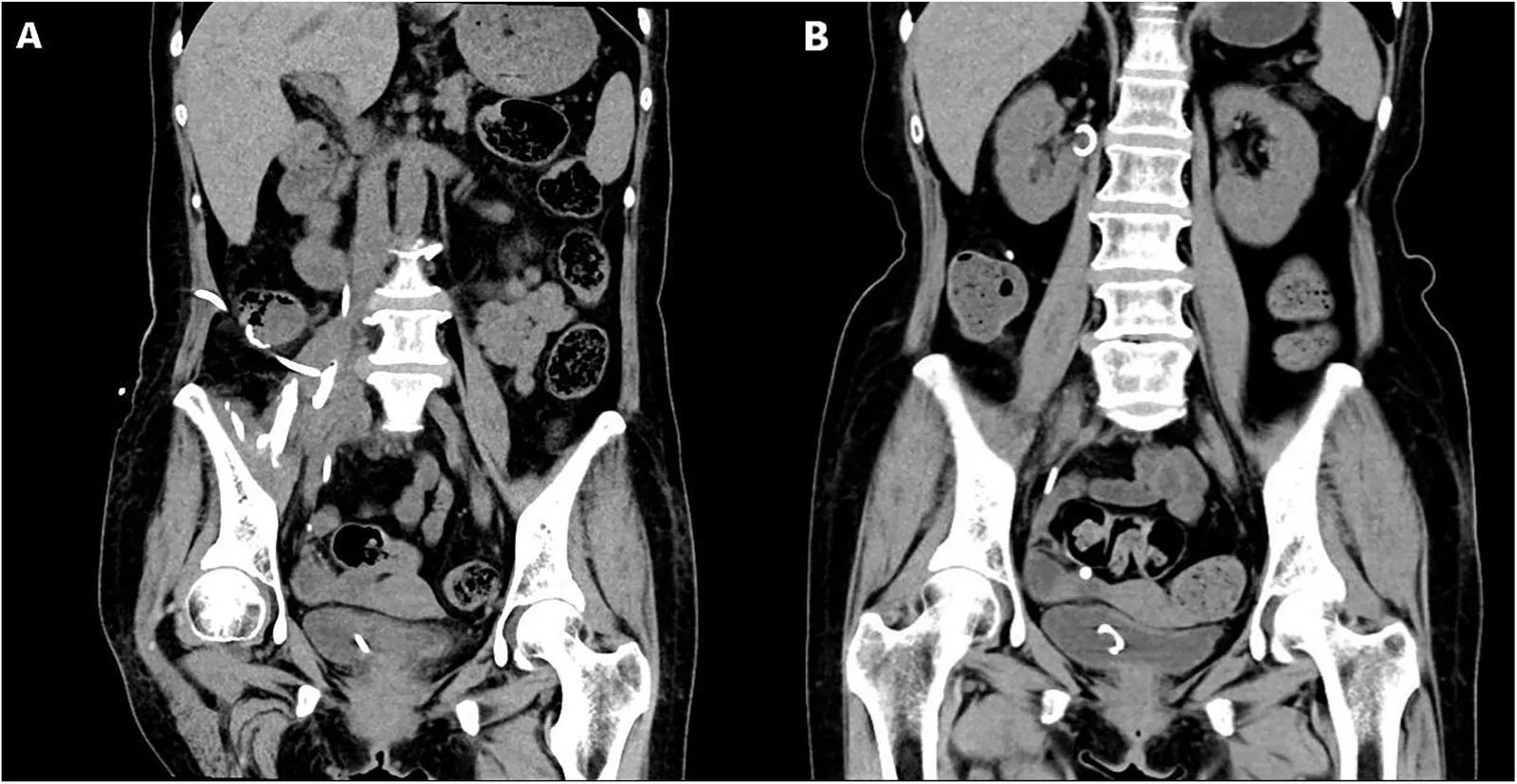
Fistulography and 1-month follow-up CT. **(A)** Fistulography performed during the procedure confirmed communication between the psoas major and iliacus abscess cavities. **(B)** One-month follow-up CT scan revealed near-normal morphology of the iliopsoas muscle and right kidney, with no residual abscess collection.

## Timeline

The timeline of the patient's clinical course is summarized in Supplementary Table 1.

## Discussion

We successfully treated a patient with a large IPA complicated by hydronephrosis caused by ureteral stent migration. Dual percutaneous drainage combined with stent exchange achieved a favorable clinical outcome. IPA is a rare condition: approximately 91% of patients present with back or flank pain and 75% with fever (12). Its clinical manifestations are often non-specific and insidious, with symptoms lasting from several weeks to 6 months, frequently leading to delayed diagnosis (3). Physical examination may reveal the “psoas sign”, defined as worsening lower abdominal pain during hip extension (13).

This sign occurs because the IPA lies posterior to the psoas fascia, which extends from the lumbar vertebrae to the iliopubic eminence. Hip movement irritates the iliopsoas muscle as well as the L2–L4 nerve roots supplying the psoas muscle, resulting in aggravated pain and limited hip mobility (14). The psoas muscle has an abundant blood supply, which enables hematogenous dissemination of pathogens from distant infection sites. Combined with its anatomical adjacency to vital tissues, this makes the muscle particularly vulnerable to infection (15). Typical laboratory abnormalities include leukocytosis, anemia, thrombocytosis, and elevated inflammatory markers such as erythrocyte sedimentation rate and C-reactive protein (3).

The clinical features of our patient were consistent with previous reports. She presented with fever, leukocytosis, and right hip pain aggravated by hip extension in the supine position. These findings prompted timely imaging evaluation, definite diagnosis and early treatment.

IPA is classified as primary or secondary. Primary cases develop via hematogenous or lymphatic spread of pathogens, while secondary cases arise from contiguous infection associated with Crohn's disease, appendicitis, ulcerative colitis, diverticulitis, colorectal cancer, urinary tract infection, urinary tract instrumentation, vertebral infection and osteomyelitis (16). The psoas muscle is highly susceptible to infection due to its abundant blood supply, which allows pathogens from distant lesions to seed locally, and its anatomical proximity to vital adjacent structures (2). Our patient had no classic risk factors for primary IPA, such as diabetes mellitus, HIV infection or intravenous drug use, indicating a secondary origin.

Microbiologically, *Staphylococcus aureus* and *Escherichia coli* are the most common pathogens of IPA (4), and pathogen distribution varies with the primary infection site (17). Fungal IPA is rare and linked to a poor prognosis, with *Candida albicans* being the most frequently isolated species (5). Fungal psoas abscesses usually result from hematogenous dissemination in immunocompromised individuals (5). Although the exact pathogenesis remains unclear, cutaneous or mucosal colonization is presumed to be the main route of pathogen entry (5). So far, only one case of IPA caused by *C. tropicalis* has been documented. Miloudi et al. (5) reported a 52-year-old man with no identifiable immunosuppression who presented after 1 month of right low-back pain, fever and general deterioration; investigations showed marked inflammation together with renal insufficiency, and CT revealed a right IPA. Percutaneous drainage yielded *C. tropicalis*, but no urinary or digestive source was found and the route of infection remained obscure. Empirical antibacterial therapy was administered; the patient deteriorated and died 2 days after drainage, before antifungal treatment could be started.

Our case differs in several clinically important respects. The patient was immunosuppressed by long-standing rheumatoid arthritis, sought care after only 7 days of symptoms, and had a urinary source, supported by proximal stent migration with anastomotic leakage and a drainage-fluid creatinine of 5,334 μmol/L (far above the serum range). Treatment with susceptibility-guided intravenous fluconazole, which combined with dual percutaneous drainage and stent exchange achieved complete resolution with no recurrence at 6 months. In contrast to the fatal outcome of the only previous report, these observations underscore that early imaging, prompt source control and timely species-directed therapy are decisive in this rare infection.

As reported in our case, the patient had a long history of RA and received long-term oral immunosuppressants and corticosteroids, which greatly increased her risk of fungal infection. Patients with RA are indeed at high risk of invasive fungal infections (IFIs) (18). Such susceptibility is attributed to autoimmune dysfunction, multiple comorbidities, and long-term use of immunosuppressive and immunomodulatory agents, including glucocorticoids, biological agents and targeted synthetic disease-modifying antirheumatic drugs (19). In particular, diabetes mellitus, pulmonary diseases, glucocorticoid therapy and methotrexate are well-recognized risk factors for IFIs among RA patients (20).

Given the patient's history of partial ureteral resection and ureteral stent placement, together with the recovery of *C. tropicalis* from both the abscess fluid and urine, the infection was most likely caused by extravasation of infected urine at the ureteral anastomosis following stent migration. Coronal contrast-enhanced CT demonstrated hydronephrosis and hydroureter with an intact renal contour and no perinephric fluid or stranding (Figure 1A); the abscess was located along the expected course of the distal ureter and extended into the right iliopsoas muscle. These imaging findings argue against forniceal rupture and instead favor leakage from a more distal ureteral site—most plausibly the prior anastomosis.

Because the IPA arose in the setting of prior intestinal oncologic surgery, a gastrointestinal origin of infection was considered; however, several findings argue against it. First, the aspirated drainage fluid had a creatinine concentration of 5,334 μmol/L—far above the normal serum range (44–106 μmol/L) and within the urinary range—directly documenting urinary extravasation despite the absence of demonstrable contrast extravasation on CT. Second, the continuous pale-yellow drainage (400–600 mL/day) ceased completely immediately after ureteral stent exchange, a temporal relationship not expected for a bowel-origin collection. Third, no enteric bacteria typical of gastrointestinal-origin abscesses (e.g., *Escherichia coli*, *Bacteroides fragilis*) were isolated; instead, *C. tropicalis* was recovered from both the abscess fluid and urine. Fourth, CT disclosed no free air, bowel-wall thickening, or contrast extravasation from the gastrointestinal tract (Supplementary CT Video 1). To further exclude alternate sources, we note that no intravascular catheter was in place and blood cultures remained sterile, arguing against a catheter-related or hematogenous focus. Collectively, these clinical, microbiological, biochemical, and radiological findings support a urological rather than gastrointestinal origin. We postulate that proximal migration of the stent caused acute obstruction, resulting in hydronephrosis and elevated intraluminal pressure that precipitated disruption at the relatively weak anastomotic site, allowing infected urine to track along the retroperitoneal space and form the iliopsoas abscess.

IPA of genitourinary origin is extremely rare, with only a few reported cases related to pyelonephritis, renal or perinephric abscesses (21, 22). In this context, ureteral stent migration deserves specific attention. In cohorts with prolonged indwelling time, reported migration rates may exceed 25% (23); migration has also been associated with inappropriate stent length, incomplete coil formation or malposition—notably a distal curl of less than 180° or a proximal coil seated in a calyx rather than the renal pelvis—and patient anatomy (11). In this patient, proximal migration most likely reflected an inadequately formed distal curl together with ureteral peristalsis, which gradually displaced the stent over the prolonged indwelling period, although the precise mechanism could not be definitively established. Preventive strategies include appropriate stent length selection confirmed by direct imaging measurement, verification of complete coil formation at both ends, and periodic imaging surveillance of stent position with timely exchange during prolonged indwelling (10, 11).

Early diagnosis of IPA remains challenging, so detailed medical history and complete physical examination are essential to heighten clinical suspicion. Clinicians should be alert to its indolent initial presentation before progression to localized lesions, and clinical inquiry should not be restricted to symptomatic evolution alone (14). CT is the diagnostic gold standard for IPA with nearly optimal diagnostic sensitivity (24), which enabled definitive diagnosis in our patient. Magnetic resonance imaging (MRI) provides superior soft-tissue and perivertebral anatomical delineation, whereas nuclear medicine imaging facilitates detection of hidden infectious foci (15). Data on PET-CT application for IPA are limited, though one study verified its value for follow-up of tuberculous psoas abscess (25). Once an abscess is confirmed, blood culture, percutaneous abscess aspiration and tissue biopsy are mandatory (3).

No standardized therapeutic regimen for IPA has been defined to date, and concurrent ureteral stent migration further complicates clinical management. Treatment decisions rely on abscess features such as lesion size, gas formation and multiloculation (17). First-line management consists of ultrasound-or CT-guided percutaneous drainage with subsequent pigtail catheter insertion, combined with pathogen-directed antimicrobial therapy; targeted antibiotics outperform empirical regimens (3). Though bedside percutaneous drainage under local anesthesia is feasible, approximately 60% of patients experience abscess recurrence, frequently requiring repeated drainage or open surgery (26).

Open surgical drainage is reserved for patients with gas-forming or multilocular IPA, as percutaneous drainage yields unsatisfactory cure rates and elevated mortality in such complicated lesions (7, 8). Nevertheless, open debridement carries substantially higher surgical invasiveness. Previous studies have demonstrated that single-agent antibiotic therapy is rarely curative for IPA larger than 3.5 cm in maximal diameter, or when coronal and axial CT dimensions surpass 5.0 and 2.3 cm separately (27). In our patient, no intralesional gas was identified on imaging, yet the abscess exceeded the aforementioned cut-off values longitudinally and transversely; accordingly, conservative antibiotic monotherapy was abandoned. Despite multilocular morphology of the lesion, minimally invasive percutaneous drainage was prioritized over open surgery.

Patients with secondary IPA usually require prolonged hospitalization for treatment of underlying diseases (15). Recurrence is commonly attributed to incomplete drainage or insufficient antibiotics leading to persistent infection (15). After IPA drainage in this patient, right ureteral stent exchange was conducted. Although initial percutaneous drainage and pus aspiration were completed successfully, follow-up CT revealed a residual collection in the right iliacus space, prompting placement of a second catheter. Fistulography confirmed communication between the psoas and iliacus cavities; the two indwelling catheters thereby permitted concurrent irrigation and drainage of both compartments, which likely contributed to the rapid symptom resolution observed within 3 days. This case illustrates that, for multilocular or communicating IPA, a single drain may prove insufficient and a second percutaneous access should be considered when residual disease is identified on surveillance imaging. Due to possible malignant contamination of the abscess tract, long-term intensive follow-up for local recurrence is essential for patients with colorectal cancer-related IPA (17).

This case report has several limitations. First, as a single-case report, the findings may not be generalizable to all patients with IPA. Second, the exact mechanism of ureteral stent migration could not be definitively confirmed, as stent position was assessed only by CT imaging. Third, the precise site of urinary extravasation was not confirmed by retrograde pyelography or delayed excretory-phase CT urography; the anastomotic origin is therefore inferred from the combined clinical, microbiological, and anatomical imaging findings rather than directly demonstrated. Nevertheless, this report has notable strengths: it describes a rare etiology of IPA secondary to ureteral stent migration, illustrates the utility of dual-tube percutaneous drainage and highlights the importance of considering fungal pathogens in immunocompromised patients presenting with IPA.

## Conclusion

Iliopsoas abscess secondary to ureteral stent migration is a rare but serious complication, particularly in immunocompromised patients such as those on long-term immunosuppression for rheumatoid arthritis. In the present case, dual ultrasound-guided percutaneous drainage combined with ureteral stent exchange achieved a favorable outcome. Clinicians should maintain a high index of suspicion for infectious complications in patients with persistent or worsening complaints after ureteral stenting, and pursue early imaging and timely source control to prevent severe septic sequelae.

## Data Availability

The original contributions presented in the study are included in the article/Supplementary Material, further inquiries can be directed to the corresponding authors.
