# Supplementary material for: A large iliopsoas abscess due to migration of ureteral stent: a case report
Source: Front Surg. 2026 Sep 29;13:1975183. doi: 10.3389/fsurg.2026.1975183 (PMC13658198; doi:10.3389/fsurg.2026.1975183)
Supplement: Supplementary file 1 [file Table1.docx]

**Supplementary Table 1**

Timeline of the patient's clinical course.

| **Time** | **Event** |
| --- | --- |
| 6 months before admission | Colon cancer surgery complicated by partial ureteral resection and ureteral stent placement |
| 7 days before admission | Onset of intermittent right lumbar pain and fever |
| Day 0 (admission) | Presented to the emergency department with fever (39.3°C), hypotension (94/71 mmHg) and tachycardia (100 beats/min). Examination revealed right costovertebral angle tenderness, right lower quadrant tenderness and a positive psoas sign (inability to extend the right hip while supine). Laboratory tests showed leukocytosis (13.71 × 10⁹/L), anemia (Hb 75 g/L), thrombocytopenia (105 × 10⁹/L) and elevated C-reactive protein (>200 mg/L); urinalysis showed pyuria (567.3/μL) and hematuria (38.3/μL). Blood cultures were sterile and no intravascular catheter was in place. Contrast-enhanced abdominal CT demonstrated right iliopsoas muscle swelling with a spindle-shaped collection, right hydronephrosis, and proximal migration of the ureteral stent. empirical cefoperazone-sulbactam 1.5 g every 12 h started |
| Day 1 | Ultrasound-guided percutaneous drainage of the psoas major abscess with placement of an 8-French pigtail catheter; drained fluid sent for culture |
| Day 3 | Abscess fluid culture (reported 2 days after drainage) and urine culture (sent on admission) both yielded *Candida tropicalis*; drainage output was 400–600 mL/day of pale-yellow fluid with a creatinine concentration of 5334 μmol/L (serum reference range 44–106 μmol/L), consistent with urine. Empirical antibacterial therapy was discontinued and targeted intravenous fluconazole 400 mg once daily was initiated according to the antibiogram (Supplementary Table 2) |
| Day 5 | Right ureteral stent exchange performed; pale-yellow drainage ceased completely, confirming urine leakage from the ureteral anastomosis |
| Day 10 (approx.) | Right iliac tenderness and restricted hip motion persisted; follow-up CT showed reduction of the psoas major abscess but a new intermuscular abscess in the right iliacus muscle. A second 8-French pigtail catheter was inserted via a lateral iliac approach under ultrasound guidance; fistulography confirmed communication between the two cavities; dual-cavity irrigation with dilute iodine solution was initiated |
| Day 13 (approx.) | Right iliac symptoms resolved |
| Day 19 (approx.) | Drainage tubes removed after purulent discharge resolved; patient discharged without further complications. Intravenous fluconazole had been administered for 2 weeks in total and was discontinued 2 days before discharge, with no oral antifungal step-down prescribed |
| 1 month post-discharge | Follow-up CT showed near-normal morphology of the iliopsoas muscle and right kidney, with no residual abscess collection |
| 2 months post-discharge | Ureteral stent removed |
| 6 months post-discharge | Patient remained recurrence-free |
