# Supplementary material for: A large iliopsoas abscess due to migration of ureteral stent: a case report
Source: Front Surg. 2026 Sep 29;13:1975183. doi: 10.3389/fsurg.2026.1975183 (PMC13658198; doi:10.3389/fsurg.2026.1975183)
Supplement: Supplementary file 2 [file Table2.docx]

**Supplementary Table 2. Antifungal susceptibility of the *Candida tropicalis* isolate(s) recovered from the drained abscess fluid and urine.**

Minimal inhibitory concentrations (MIC) and interpretations according to CLSI M27/M60 documents, determined by broth microdilution (instrument-based). The drained abscess fluid was cultured by direct plating under 5% CO_2_, and species identification was performed by matrix-assisted laser desorption/ionization time-of-flight mass spectrometry (MALDI-TOF MS). MIC values correspond to the isolate recovered from the drained abscess fluid; the urine isolate was reported as the same species with an identical susceptibility profile.

| **Antifungal agent** | **MIC (µg/mL)** | **Interpretation (S/I/R)** | **Method / breakpoint source** | **Breakpoints (S / [I] / R) µg/mL** |
| --- | --- | --- | --- | --- |
| Fluconazole | 8 | S (susceptible) | Broth microdilution (instrument-based); breakpoints as adopted by our laboratory in 2024 | ≤8 / 16–32 / ≥64 |
| Voriconazole | 0.5 | S (susceptible) | Broth microdilution (instrument-based); breakpoints as adopted by our laboratory in 2024 | ≤1 / – / ≥4 |
| Amphotericin B | ≤0.5 | S (susceptible) | Broth microdilution (instrument-based); breakpoints as adopted by our laboratory in 2024 | ≤1 / – / ≥4 |
| Flucytosine | ≤4 | S (susceptible) | Broth microdilution (instrument-based); breakpoints as adopted by our laboratory in 2024 | ≤4 / – / ≥32 |
| Itraconazole | ≥4 | R (resistant) | Broth microdilution (instrument-based); breakpoints as adopted by our laboratory in 2024 | ≤0.125 / – / ≥1 |
